# PK/PD of single-dose amikacin in emergency department patients with severe sepsis/shock: should we apply the ICU-based higher loading dose?

**DOI:** 10.1186/cc14198

**Published:** 2015-03-16

**Authors:** S De Winter, J Wauters, E Van Wijngaerden, W Peetermans, P Annaert, J Verhaegen, JB Gillet, D Knockaert, I Spriet

**Affiliations:** 1 University Hospitals Leuven, Belgium; 2 Catholic University Leuven, Belgium

## Introduction

Studies in the ICU showed that a single amikacin dose of ≥25 mg/kg should be used in conditions of increased distribution volume (Vd) such as severe sepsis/shock [1]. However, no data are available for emergency department (ED) patients in the early phase of sepsis/septic shock. The purpose of this study was to determine whether a single amikacin dose of 25 versus 15 mg/kg results in PK/PD target attainment for ED patients.

## Methods

ED patients with severe sepsis/shock were randomly treated with a single amikacin dose of 25 versus 15 mg/kg. Blood samples were collected at +1 (peak), +6 hours and +24 hours (trough) after the start of infusion. Primary outcome was PK/PD target attainment defined as a peak/MIC >8, corresponding with both actual MIC values documented from isolated pathogens, as well as EUCAST susceptibility breakpoints for Enterobacteriaceae and *P. aeruginosa*; that is, 8 mg/l. Noncompartmental analysis was used to calculate PK parameters.

## Results

During a study duration of 20 months, 50 patients were enrolled in each dosing regimen resulting in 100 peak concentrations, 92 and 88 +6 hours and +24 hours concentrations respectively. Target attainment using local MIC values (median 2 mg/l, documented in 56 isolated Gram-negative pathogens) was achieved in 95% in both groups (*P *= 0.98). Using EUCAST susceptibility breakpoints, the target was attained in 76% versus 40% in the 25 versus 15 mg/kg group, respectively (*P *< 0.0001). Single-dose PK parameters are displayed in Table 1 and compared with the ones reported in the ICU [1].

**Table 1:** 

PK parameter	15 mg/kg ED	25 mg/kg ED	25 mg/kg ICU
Peak (mg/l)	58(47 to 70)^a^	91(72 to 105)^a^	73(62 to 90)
Trough (mg/l)	6(3 to 12)	5(3 to 15)	7(2 to 15)
Vd (l/kg)	0.3(0.3 to 0.5)	0.4(0.2 to 0.6)	0.4(0.3 to 0.5)
Cl (ml/minute/kg)	1.6(1 to 2.3)^a^	2.2(1.4 to 3)^a^	1.9(1.3 to 3.5)

Cl, clearance. ^a^Mann-Whitney *U *<0.05 15 versus 25 mg/kg ED patients.

## Conclusion

The EUCAST-based PK/PD target was only attained in 76% of patients treated with 25 mg/kg. However, in contrast to ICU patients, the majority of ED patients are treated for community-acquired infections, so MIC values are significantly lower than the EUCAST susceptibility breakpoints, warranting PK/PD target attainment in both 25 and 15 mg/kg dosing regimens when local epidemiology is taken into account.
